# Biodiversity in tropical rainforests: *Calleida* Dejean, 1825 at the BIOLAT Biological Station, Rio Manu, Peru, with descriptions of seven new species (ColeopteraCarabidae, Lebiini). Part 1

**DOI:** 10.3897/zookeys.1044.64082

**Published:** 2021-06-16

**Authors:** Achille Casale

**Affiliations:** 1 University of Sassari, Italy (Zoology). Private: Corso Raffaello 12, 10126 Torino, Italy University of Sassari Sassari Italy

**Keywords:** Biodiversity, canopy specialists, Neotropics, Madre de Dios

## Abstract

A monographic contribution is presented on the species of the genus *Calleida* Dejean, 1825 at the BIOLAT Biological Station, Rio Manu, Pakitza, Peru, sampled by Terry Erwin and his co-workers. The following seven new species are described: *C. solitaria***sp. nov.**, *C. manuensis***sp. nov.**, *C. anomala***sp. nov.**, *C. demathani***sp. nov.** (type locality: Peru, Tarapoto, but sampled also at Rio Manu), *C. erwini***sp. nov.**, *C. marginithorax***sp. nov.**, and *C. maxima***sp. nov.** Relationships of each species are discussed, and a preliminary survey is presented of the genus *Calleida* in Peru.

## Introduction

As recalled by Erwin (1983a, b), tropical rain forest canopies are “the last biotic frontier”. The upper Amazon basin in South America, forming an arc included in the Eastern territories of Ecuador, Peru, Bolivia, and western Brazil, is currently cited as one of the main centers of biodiversity in the world (Wilson 1992). In this area, beetles of the family Carabidae represent one of the better-examined insect groups; even so, there remain thousands of undescribed species, and many lacking data regarding habitat choice and life history (Erwin 1991). Calleidina are a very diverse assemblage of genera and species of CarabidaeLebiini distributed on all continents, with their highest diversity in tropical regions. Most of them are specialized arboreal or subarboreal beetles, tied to foliage in forest canopies (Casale 1998). Several years ago, I commenced revision of the genus *Calleida* Dejean, 1825 in the former (and widest) sense, with the aim to make evident the phylogenetic relationships among taxa, many of which are undescribed, from the different parts of the world. For this work-in-progress, I received help and material from many colleagues, friends, institutes, and museums.

Concerning the present contribution, I am particularly indebted to Terry Erwin (Department of Entomology, National Museum of Natural History, Smithsonian Institution, Washington), a well-known specialist not only of carabids, but more in general of the biological diversity of Arthropoda in tropical forests. Amongst the many Neotropical areas explored by Erwin in many years of research, one in particular revealed the outstanding diversity of the insect fauna of canopies in tropical rainforests: the Manu Reserve Zone, at the southern edge of Manu National Park, in the Amazon basin of south-eastern Peru (Erwin 1991). The present contribution, in two parts, will be dedicated to the species of *Calleida* sampled by Terry Erwin and his co-workers of the BIOLAT Program, with additional notes on some representatives of the genus in Peru.

### The genus *Calleida* Dejean, 1825 in Peru: a survey

Peru is a South American country that includes a great variety of environments, with its 1,285,215 square kms, due to its privileged geographical position between the Equator and the 20^th^ Parallel South, the occurrence of some of the highest peaks of Southern America (Uascarán, 6768 m a.s.l.), an important fluvial system mostly tributary of the Amazon basin, and the presence of tropical rain and cloud forests on both the Pacific and Amazon sides of the Andes. However, as in other tropical areas, and in spite of many contributions dedicated to the knowledge of the Peruvian fauna over many years (for example, the historical German expedition in 1936: see Liebke 1941), the insect fauna of Peru is little known at present. Concerning the Calleidina, the recent catalogue by Lorenz (2005) lists more than 170 Neotropical species in the genus *Calleida*. However, only fourteen of them were cited from Peru in the current literature. Chaudoir, in his monograph (1872), cited *C. alcyonea* Erichson, 1847 (possibly synonym of *C. smaragdula* Reiche, 1842), *C. prolixa* Erichson, 1847, *C. tibialis* Brullé, 1837 and *C. mniszechi* Chaudoir, 1852 from Peru. Later, Chaudoir (1877) described *C. viridiaurea* from this country. More recently Liebke (1935, 1941) described *C. horni*, *C. jeanneli* and *C. titschacki* from Peru. Finally Casale (in Desender et al. 2002) described *C. migratoria* from western Peru (Piura, Olmos 1800 m a.s.l.), a species introduced and now widely spread in Galapagos islands, politically belonging to the Ecuador republic, and also cited *C. haematodera* Chaudoir, 1872 for the first time from Peru.

A more recent, important contribution by Erwin et al. (2015) listed 721 species of carabids described so far from Peru, with eight *Calleida* species included. In recent years, I was able to examine several new *Calleida* species from different areas of Peru, but the present contribution dedicated to the Rio Manu area is particularly significant, showing how many close or sibling species can coexist in the same, reduced, surface area in tropical rainforests, as an example of adaptive radiation in macro- and micro-habitats (see Erwin and Pogue 1988). Furthermore, the sampling by Erwin is particularly exceptional for other reasons: first, it arises from a long-term program of research in a geographically defined surface (many species described so far from Peru having the mere type locality “Peru”); second, the area has been described in detail in all aspects from the geographical and ecological points of view; third, every sampled individual bears precise data and methods of collection on its label (arboreal species, coordinates).

## Materials and methods

The following data come from a series of more than 200 examined specimens from the BIOLAT Biological Station, Rio Manu, Pakitza, Peru, at the southern edge of Manu National Park, in the upper Amazon basin of Peru. Additional *Calleida* specimens were examined, including type series important for the correct identification of the Peruvian material.

Male and female genitalia were dissected, dehydrated in ethanol, cleared in cold KOH, examined, and illustrated using standard techniques before their definitive inclusion on microscope slides. Line drawings were made using a camera lucida attached to microscopes Wild M-5 and M-3, and a microscope Leitz Orthoplan. For female specimens, in this preliminary contribution only features of the ovipositor (stylomeres) are illustrated for two species, a new species included known from one only individual. A key for identification of Peruvian *Calleida* species will be provided in a next contribution.

### Taxonomic treatment and morphological terms

The genus *Calleida* is here treated in its widest sense, as a member (following the rules of ICZN) of the family Carabidae, Subfamily Lebiinae, Tribe Agrini, Subtribe Calleidina. This group, in the narrow sense of Casale (1998, 2007), Casale and Shi (2018), Ball and Hilchie (1983), Ball and Bousquet (2001) (= Callidides of Chaudoir 1872, pars; Calleidini of Jeannel 1949, pars; *Callidina* of Habu 1967, pars; Callidini of Basilewsky 1984; Agrina Calleidida group of Erwin 2004) are a monophyletic unit, markedly distinct for both adult and larval features, and the morphological characters of male and female genitalia. This fact was also confirmed by a phylogeny of CarabidaeHarpalinae recently presented by Ober and Maddison (2008). The genus *Calleida* is treated in the wider sense, characterized by a set of morphological characters which distinguish it from other Neotropical genera of Lebiini (see Erwin 2004). The median lobe of the aedeagus is synonymous with the phallus of some authors. The proximal gonocoxite 1 and the more distal gonocoxite 2 (in the sense of Liebherr and Will 1998) are synonyms of stylomere 1 and stylomere 2 of authors, respectively.

### Acronyms

**EL/EW** ratio Length of Elytra, as linear distance from the basal ridge to the apex, measured along the suture/maximum Width of Elytra;

**L** overall Length, from apex of mandibles to apex of elytra, measured along the suture;

**PL/PW** ratio Length of Pronotum, as linear distance from the anterior to the basal margin, measured along the midline/maximum Width of Pronotum, as greatest transverse distance;

**TL** body Total Length, from the anterior margin of clypeus to the apex of elytra, measured along the suture.

### Collections and depositories

**CCa** A. Casale, c/o University of Sassari, Sassari and Torino (private collection) (Italy);

**MNHN**Muséum National d’Histoire Naturelle, Entomologie, Paris (France);

**NMNH**National Museum of Natural History, Department of Entomology, Washington, D.C. (U.S.A.) (holotypes held in trust at NMNH for transfer to Museo de Historia Natural San Marcos, Lima, Peru);

**NHMUK**National History Museum, London (UK).

### The area

**Figure 1.**
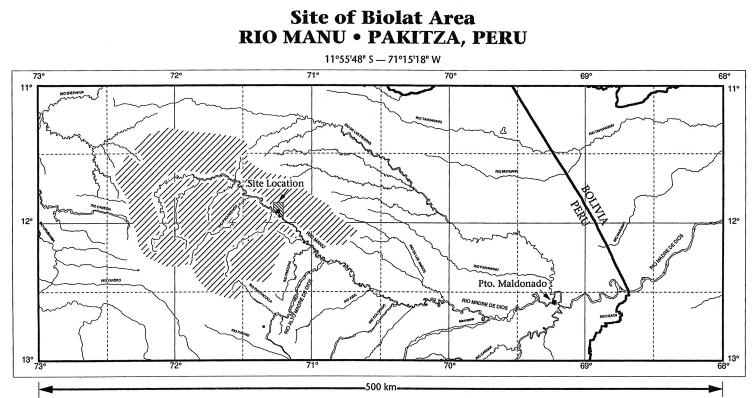
Site and map of the Biolat area at Rio Manu, Peru (after Erwin 1991).

**Figure 2.**
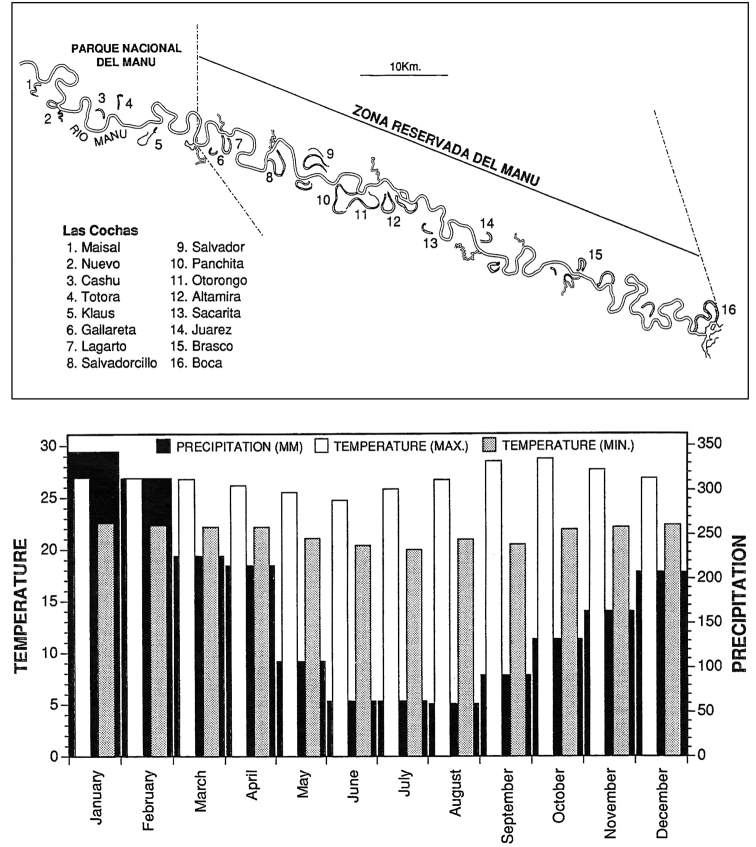
Map, rainfall, and temperatures of Rio Manu basin (after Erwin 1991).

**Figure 3.**
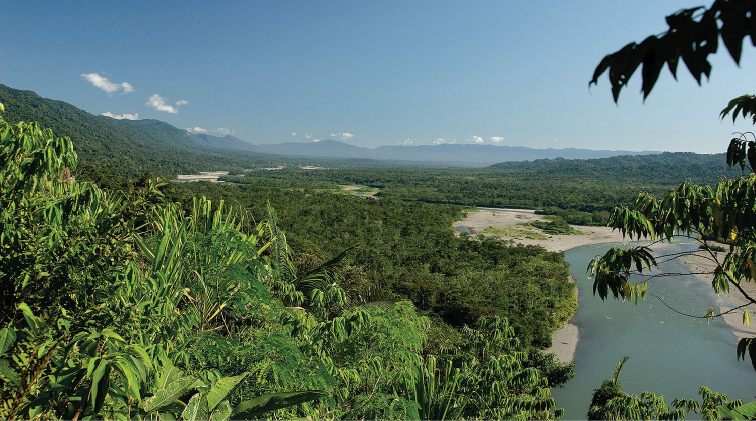
Rainforest at the Rio Manu basin (Photo Corey Spruit, Manu National Park).

The area and methods of sampling and mapping have been described in detail by Erwin (1991), after a survey performed from September 1987 to November 1990: the Reserved Zone of Manu (south-eastern Peru, at the frontier with Bolivia) is in the broad Manu River Valley between Manu National Park and the Rio Madre de Dios and includes, in the northern part, a narrow strip of upland ridgetop forest on both sides of the valley (Figs 1–3). The Vigilante Post, Pakitza, and the associated BIOLAT Biological Station (356 m) are approximately half way up the Rio Manu from its mouth at the Rio Madre de Dios, ca. 65 km (river distance), and ca. 32 km from the Andes, at 11°56'47"S, 071°17'00"W. The high plant diversity in the area includes more than 1850 species (Erwin 1991). Erwin (1991) illustrated both the habitats at the BIOLAT Station, which includes non-flooded forest, seasonally-flooded forests, open areas, and other vegetative habitats and microhabitats for Carabidae within or across habitats at Pakitza (canopy layer, subcanopy layer, undercanopy layer, ground cover layer, soil and gravel layer), with a list of sampling methods for each habitat. Furthermore Erwin (1991, 1996) provided a checklist and keys to all tribes and genera of carabids found at this site, with excellent drawings to aid future workers.

## Descriptions of new *Calleida* species

### Calleida solitaria sp. nov.

Taxon classificationAnimaliaColeopteraCarabidae

E633EE75-AE01-535C-A730-1C970C8844BB

http://zoobank.org/2C830D71-E6EF-4842-8103-E15383F00626

Figures 4 , 8

#### Type locality.

Peru, Madre de Dios: Rio Manu, Pakitza, 356 m 12°07'S, 70°58'W.

#### Type material.

***Holotype***, male: “PERU, MADRE DE DIOS, Pakitza, 14–20 Oct 90 T.L. Erwin Coll. 12°07'S, 70°58'W, at lab lights”, “BIOLAT/COLE 000006017” (NMNH)

#### Specific epithet.

Feminine adjective, from the Latin *solitarius, -a* (lonely), which indicates that this new species is known from a single individual only, flying at night.

**Figure 4.**
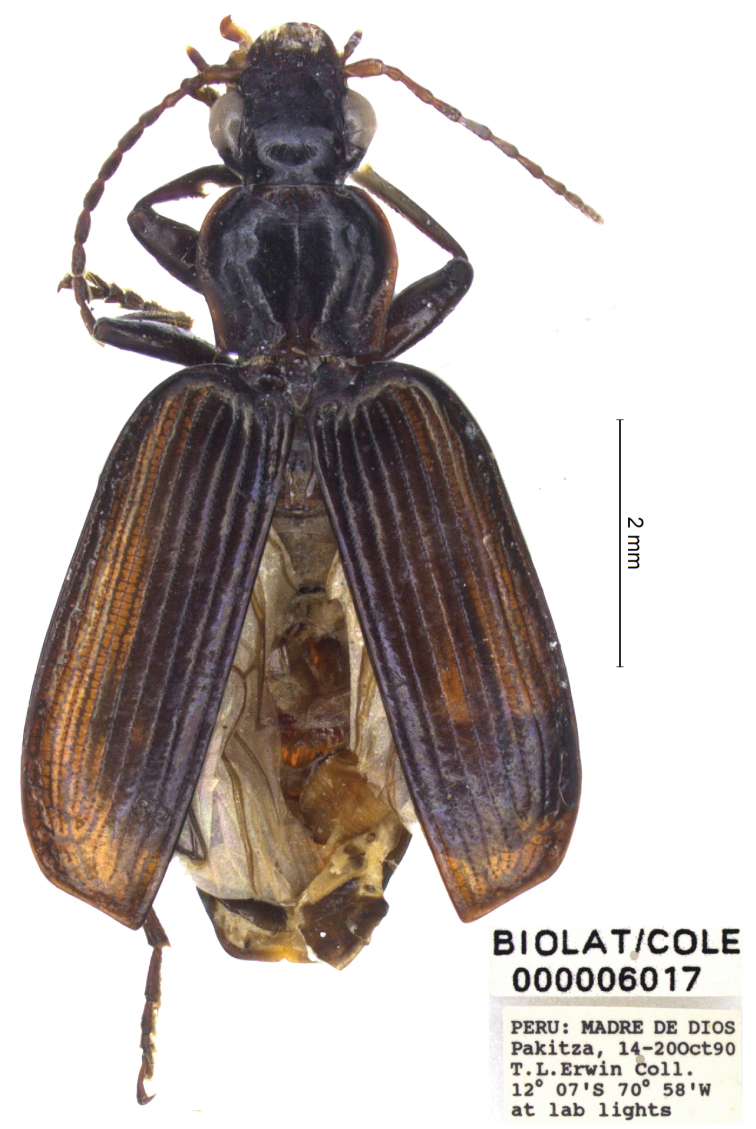
*Calleida solitaria* sp. nov., male holotype, habitus.

**Figure 5.**
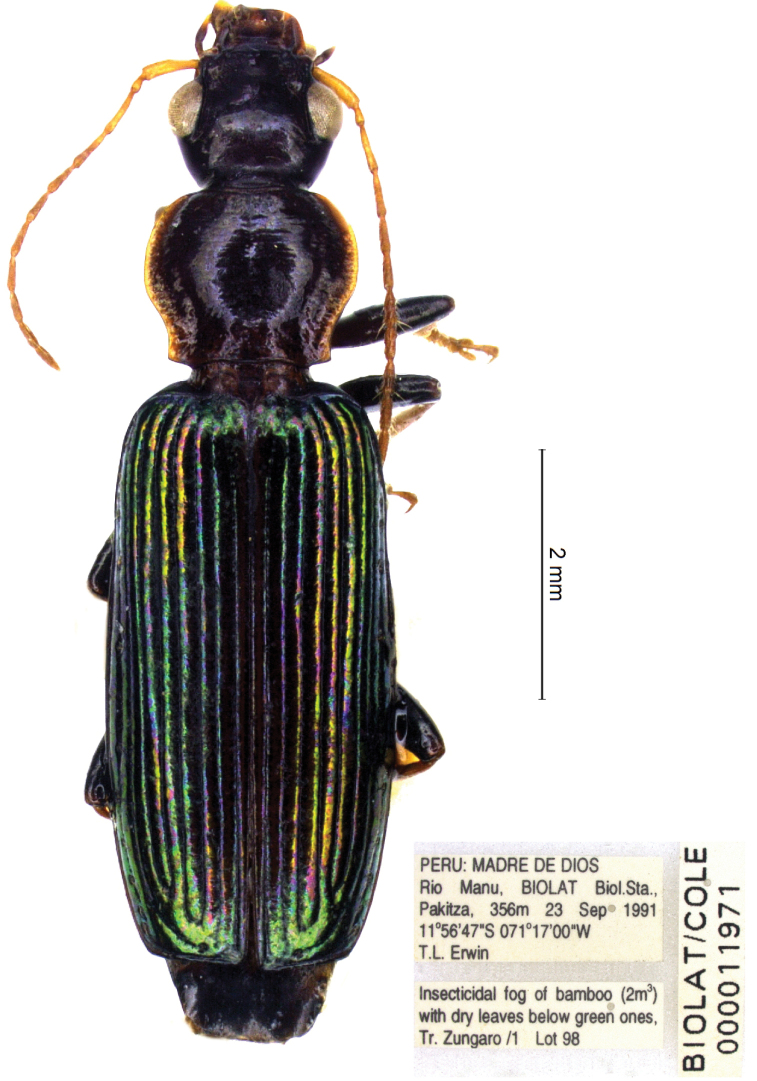
*Calleida manuensis* sp. nov., male holotype, habitus.

#### Diagnosis.

With the character states of the genus *Calleida* in the narrow sense, but characterized by the peculiar combination of the following morphological features: small-medium sized (TL: mm 7.1); body brown, darkened on head and disc of pronotum; antennae, tibiae, tarsi, and abdominal segments rufous. Pronotum cordiform; elytra elongate, depressed, beaded at apex, with deep and deeply punctate striae. Abdominal sternum VII with one seta on each side in males. Male genitalia as in Fig. 8. Female unknown.

#### Description.

General features as in Fig. 4. Small-medium sized: L: 7.6 mm; TL: 7.1 mm.

***Color***: head dark brown, without reddish spots on frons; antennomeres dark rufous. Prothorax and pterothorax brown; pronotum dark reddish at sides. Elytra brown, with slight metallic bronze reflection; tibiae, tarsi, and abdominal segments dark rufous.

***Luster and microsculpture***: head and pronotum moderately glossy, with generally effaced cuticular microlines, hardly visible as reticulate meshes; elytra moderately glossy, with cuticular microlines evident in form of transverse pattern.

***Head***: wide, transverse, with evident, deep neck constriction; genae very swollen, contiguous with the posterior margin of eyes; frontal furrows deep, connected posteriorly with the supra-orbital keels; eyes very large and prominent; two supraorbital setae on each side.

**Figures 6, 7.**
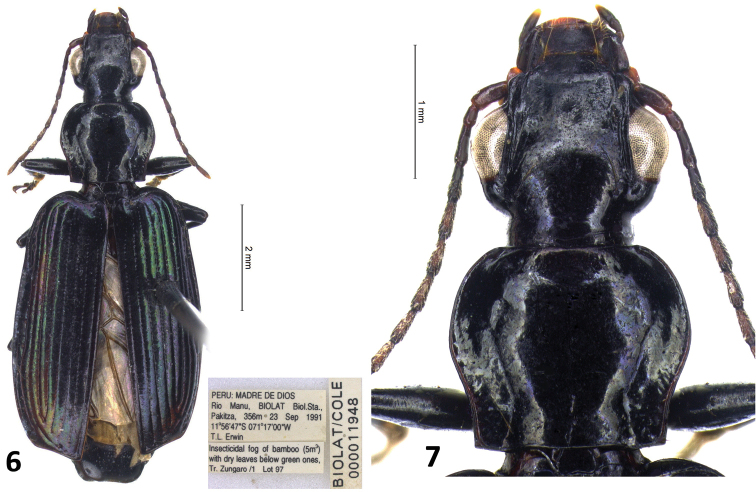
*Calleida anomala* sp. nov. **6** male holotype, habitus **7** idem, head and pronotum.

***Prothorax***: transverse-cordiform (ratio PL/PW: 8.82), with lateral margins moderately sinuate in the posterior third and constricted to the base. Lateral furrows narrow, punctate; lateral reflection slight. Disc convex, with superficial transverse wrinkles. Median furrow deep, reaching the posterior margin. Anterior angles rounded, not prominent; basal angles obtuse but evident. Base almost straight, beaded. One paramedial seta and one basolateral seta on each side present.

***Elytra***: elongate (ratio EL/EW: 1.8), depressed, slightly widened at the apical third; striae deep, deeply punctate; intervals slightly convex. Post-humeral sinuation inconspicuous, pre-apical callosity absent. Apex beaded, oblique, with the outer angle fully rounded and sutural angle prominent. Interval 3 with two discal and one apical setiferous puncture; umbilicate series of 14 large foveate punctures along stria 8, widely interrupted in the middle.

***Hind wings***: fully developed.

***Legs***: slender. Metatarsomeres 1–3 superficially grooved dorsally; metatarsomere 4 deeply bilobed, its lobes long and narrow. Tarsal claws denticulate, each with five long teeth on the inner side.

***Abdominal sterna***: sternum VII with one seta on each side in males (unknown, but probably two setae on each side in females).

**Figures 8–10.**
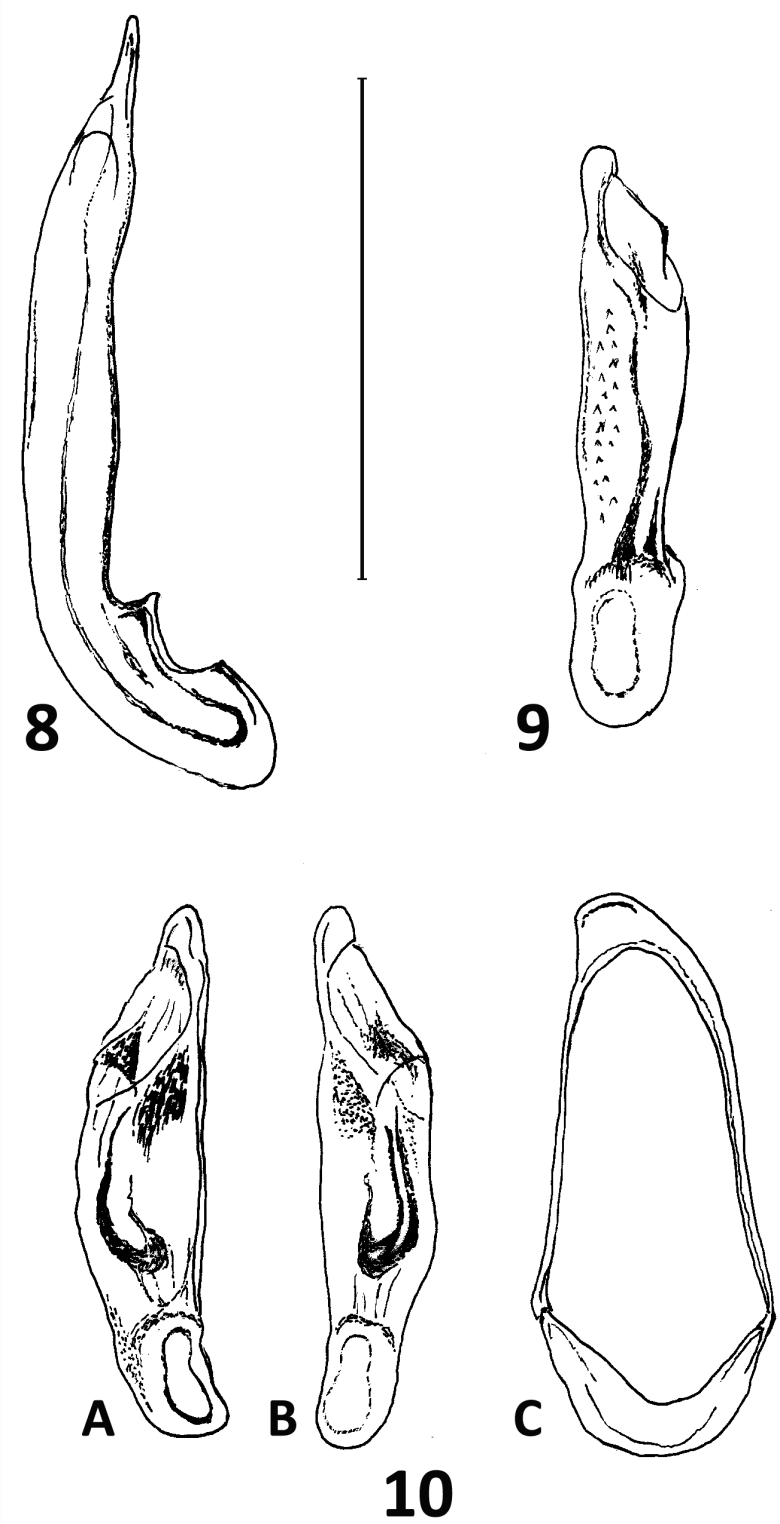
Median lobe of aedeagus and abdominal segment IX of *Calleida* species **8***C. solitaria* sp. nov., male holotype **9***C. manuensis* sp. nov., male holotype **10***C. anomala* sp. nov., male holotype **A** left lateral aspect **B** dorsal aspect **C** abdominal segment IX. Scale bar: 1 mm.

***Male genitalia***: median lobe of aedeagus (Fig. 8) very elongate and slender, with basal lobe markedly differentiated. Endophallus with a long flagellum, twisted at base.

***Female genitalia***: unknown.

#### Geographical distribution and habitat.

Known so far from the type locality only. It is probably a nocturnal forest-dwelling species at lower altitudes. The single male individual of this taxon was obtained in October, at the lights of the Biolab in Pakitza.

#### Relationships.

For its basic morphological features of shape, size, and characters of the median lobe of aedeagus and endophallus, this species seems to be close to the species of the *cordicollis* (= *decora*) species group in the sense of Casale in Desender et al (2002), from which it is markedly distinct by the different pattern of color on the dorsal side (mostly metallic in the *cordicollis* group), and by the different number of setae on the abdominal sternum VII (two in male in *C. solitaria*, three in species of the *cordicollis* group).

### Calleida manuensis sp. nov.

Taxon classificationAnimaliaColeopteraCarabidae

83B76E68-24BF-592B-9F14-E5BD6039D816

http://zoobank.org/F1E498D2-AF46-490A-BA4A-B9965F52E04A

Figures 5 , 9

#### Type locality.

Peru, Madre de Dios, Rio Manu, BIOLAT Biological Station, Pakitza, 356 m.

#### Type material.

***Holotype***, male: “PERU, MADRE DE DIOS, Rio Manu, BIOLAT Biol. Sta., Pakitza, 356 m 23 Sep 1991 11°56'47"S, 071°17'00"W, T.L. Erwin”, “BIOLAT/COLE 000011971”, “Insecticidal fog of bamboo with dry leaves below green ones. Tr. Zungaro/1 Lot 98” (NMNH). ***Paratypes*** (NMNH, CCa): 1 ♀, same locality and data as holotype, BIOLAT/COLE 000011943, Insecticidal fog of bamboo with dry leaves below green ones. Tr. Zungaro/1 Lot 97; 1 ♂, same locality and data as holotype, BIOLAT/COLE 000011972; 1 ♂ same locality as holotype, 26 Sep 1991, T.L. Erwin, Insecticidal fog of bamboo at 4 m with lots of dry leaves, stems, dense green above Tr. Zungaro/3 Lot 122, BIOLAT COLE 000012118; 1 ♂, same locality as holotype, 11 Oct 1991, T.L. Erwin & M.G. Pogue, Insecticidal fog of Astrocaryum dry leaves (6 fronds) 3.5 Weight Tr. Caña Brava/ 7.5 Lot 240, BIOLAT COLE 000013826; 2 ♀♀, same locality as holotype, 11 Oct 1991, T.L. Erwin & M.G. Pogue, Insecticidal fog of Guadua green dense cover with 0.3 m dead broad leaves under at 2.5 m Tr. R1/23–25 Lot 245, BIOLAT COLE 000013920.

#### Specific epithet.

Geographical epithet, from the type locality of this species, the Rio Manu in Peru.

#### Diagnosis.

With the character states of the genus *Calleida*, but from the closest Neotropical species markedly characterized by the peculiar combination of the following morphological features: small-medium sized (TL: mm 7.5–8.0); body very elongate, slender and narrow; head ovate-elongate, with genae swollen and abruptly constricted at the neck; pronotum cordiform, with lateral margins deeply sinuate in the posterior third, disc transversely wrinkled and basolateral seta absent; elytra parallel sided, deeply striate, beaded at apex. Head and pronotum dark brown reddish (yellow-rufous in some individuals), contrasting in color with the dark metallic green or cupreous green elytra; antennae reddish yellow; legs rufous, with darkened, blackish brown femora. Abdominal sternum VII with two setae on each side in males (one only, exceptionally, in one examined individual), three (exceptionally four, on one side) setae in females. Male genitalia as in Fig. 9.

#### Description.

General features as in Fig. 5. Small-medium sized: L: 8.0–8.5 mm; TL: 7.5–8.0 mm.

***Color***: head, prothorax and pterothorax concolorous brown reddish (rufous in teneral individuals); abdomen brown blackish (rufous in teneral individuals); antennae, trochanters, tibiae, and tarsi uniformly rufous; femora darkened, brown reddish to blackish; elytra dark metallic green or cupreous green, in some individuals with golden reflections.

***Luster and microsculpture***: Head and pronotum glossy, cuticular microlines mostly effaced; elytra moderately glossy, with mesh pattern transverse, and evident microlines.

***Head***: elongate, ovate, with deep neck constriction, evident also on the dorsal side; genae swollen, markedly curved and abruptly constricted at the neck, not contiguous with the posterior margin of eyes which are longer than genae and very prominent; frons smooth; frontal furrows superficial, deeply and densely punctate in front; two supraorbital setae on each side.

***Prothorax***: transverse-cordiform (ratio PL/PW: 0.78), with lateral margins deeply sinuate in the posterior third and constricted to the base. Lateral grooves wide, flattened, punctate; lateral reflection moderate, evident in the basal fourth. Disc convex, with dense, deep transverse wrinkles and sparse, small punctuations. Anterior angles rounded, fully effaced; basal angles obtusely marked, slightly prominent outside. Base beaded, oblique at the extreme lateral margins. One paramedial seta on each side present; basolateral seta absent.

***Elytra***: elongate and narrow (ratio EL/EW: 2.0), sub-convex, parallel sided; striae markedly impressed, deeply punctate; intervals convex. Post-humeral sinuation very shallow, pre-apical outer callosity absent. Apex beaded. Interval 3 with two small discal and one apical setiferous punctures; umbilicate series of 13 punctures along stria 8, interrupted in the middle.

***Hind wings***: fully developed.

***Legs***: short, robust. Tarsomeres of stout form. Metatarsomeres 1–3 flattened, with shallow traces of dorsal grooves; metatarsomere 4 deeply bilobed, its lobes slightly widened and rounded at apex. Tarsal claws denticulate, each with five long teeth on the inner side.

***Abdominal sterna***: sternum VII with two setae on each side in males (exceptionally one, in one examined individual), three setae in females (exceptionally and asymmetrically four on one side in one examined individual).

***Male genitalia***: median lobe of aedeagus (Fig. 9) small, short, tubular, moderately curved; apex short, rounded distally. Endophallus with a short, curved, and undulate flagellum, not twisted at base, and a small, basal copulatory piece.

***Female genitalia***: not examined.

#### Geographical distribution and habitat.

*Calleida manuensis* is known so far from the type locality only: Peru, Madre de Dios, Rio Manu, BIOLAT Biological Station, Pakitza, 356 m a.s.l. The specimens of the type series were obtained by insecticidal fog of bamboo, *Astrocaryum* and *Guadua* in September and October.

#### Relationships.

*Calleida manuensis*, in both external features (general shape of body, disc of pronotum transversely wrinkled, and abdominal sternum VII with two setae on each side in males and three in females) and the characters state of male genitalia (endophallus with a long, bent flagellum and a small basal copulatory piece), seems to be related to *C. gounellei* Liebke, 1935 from southern Brazil and Paraguay. From the latter, it is easily distinguishable for the different pattern of color (the pronotum is mostly metallic golden-green in *C. gounellei*), ovate head (much more transverse in *C. gounellei*), and the markedly cordate pronotum (subquadrate, with lateral margins not or slightly sinuate basally in *C. gounellei*).

### Calleida anomala sp. nov.

Taxon classificationAnimaliaColeopteraCarabidae

1BF2A773-04D1-5EA1-8130-B1ACB428A18C

http://zoobank.org/0F8DEABB-92A7-40FA-819E-4F44340E1D20

Figures 6 , 10

#### Type locality.

Peru, Madre de Dios, Rio Manu, BIOLAT Biological Station, Pakitza, 356 m.

#### Type material.

***Holotype***, ♂ (NMNH): “PERU, MADRE DE DIOS, Rio Manu, BIOLAT Biol. Sta., Pakitza, 356 m 23 Sep 1991 11°56'47"S, 071°17'00"W, T.L. Erwin”, “BIOLAT/COLE 000011948”, “Insecticidal fog of bamboo (5 m) with dry leaves below green ones. Tr. Zungaro/1 Lot 97”. ***Paratypes*** (NMNH, CCa): 1 ♂, same data as holotype, BIOLAT/COLE 000011942; 1 ♂, same locality and collector as holotype, 20 Sep 1991, Insecticidal fog of scattered dry leaves in green, 1 m, Tr. Tachigali / 16.8, dissected alluvial terrace forest Lot 88; 1 ♀, same locality and collector as holotype, 22 Sep 1991, Insecticidal fog of dry leaves (2 m), alluvial terrace forest, Tr. Tachigali / 9 Lot 96, BIOLAT/COLE 000011912; 1 ♀, same locality and collector as holotype, 25 Sep 1991, Insecticidal fog of dry leaves at 2.3 m with thin bamboo canopy Tr. Pacal / 8 Lot 110, BIOLAT/COLE 000012016; 1 ♀, same locality and collector as holotype, 26 Sep 1991, Insecticidal fog of bamboo at 3–4 m green some dry leaves, Tr. Zungaro/4 Lot 117, BIOLAT/COLE 000012088; 1 ♀, same locality as holotype, 30 Sep 1991, T.L. Erwin & M.G. Pogue, Insecticidal fog of dense vine tangle plus leaves, sparse bamboo cover (3 m) Tr. Tachigali / 22 Lot 137, BIOLAT/COLE 000012344; 3♂♂, 1♀, same locality, date and collectors, Insecticidal fog of dry leaves, Swartia and bamboo overstory with some green leaves Tr. Tachigali / 23 Lot 140, BIOLAT/COLE 000012325, 12326, 12328, 12362; 3♂♂, 1♀, same locality, date and collectors, Insecticidal fog of dense dry vine tangle at 5 m next to old dead tree with some green leaves up at 15 m Tr. Tachigali / 23 Lot 141, BIOLAT/COLE 000012464, 12465, 12466, 12467; 1 ♀ (teneral), same locality and collectors, 02 Oct 1991, Insecticidal fog of bamboo-broadleaf mix at 3 m with 0.5 m dry bamboo leaves on dry culmus and vines Tr. Tachigali / 38 Lot 165, BIOLAT/COLE 000012797; 1 ♀, same locality, date and collectors, Insecticidal fog of thin bamboo over dense dry bamboo leaves 1 m at 2 m Tr. Tachigali / 38 Lot 173, BIOLAT/COLE 0000128161 ♂, same locality as holotype, 14–20 Oct 1992, 11°58'23"S, 71°15'25"W T.L. Erwin, At lab lights, Lot 289, BIOLAT/COLE 000015165; 2♂♂, 3♀♀, same locality as holotype, 23 June 1993, T.L. Erwin & F. Pfuno, Insecticidal fogging of medium-sized dry leaves at 6 m in dry vines of tree, 3 m, some light bamboo above but not tree leaves Tr. Tachigali / 22 Lot 498, BIOLAT/COLE 000018460, 18461, 18462, 18463, 18464; 1 ♀, same locality, date and collectors, Insecticidal fogging of medium-sized dry leaves at 7 m in dry vines of tree, 2 m,, some light broad leaf above but not bamboo, Tr. Tachigali / 22 Lot 500, BIOLAT/COLE 000018513; 1♂, 2♀♀, same locality, date and collectors, Insecticidal fogging of complex canopy at 20 m top, W / 9 vines, crown 7 m dia, with some dry leaves in vines at 6 m Tr. Tachigali / 20 Lot 505.

#### Specific epithet.

The specific name, *anomala*, indicates the unique features of this new species into the genus, i.e., the absence of the paramedial (anterior) seta of the pronotum, and the peculiar shape of head and pronotum.

#### Diagnosis.

With the character states of the genus *Calleida* (in the wider sense: see Materials and methods), but from the closest Neotropical species markedly characterized by the peculiar combination of the following morphological features: small-medium sized (TL: mm 7.1–8.5); body moderately elongate, depressed; head subquadrate, with genae very swollen and abruptly constricted at the neck; pronotum transverse-cordiform, with lateral margins markedly sinuate in the posterior third and paramedial seta absent; elytra slightly widened at the apical third, deeply striate, beaded at apex. Head, pronotum, underside and legs dark brown to blackish, moderately contrasting in color with the dark metallic green, blue green, blue violet, or violet elytra. Abdominal sternum VII with one seta on each side in males, two setae in females. Abdominal sternum IX as in Fig. 10C. Male genitalia as in Fig. 10A, B.

#### Description.

General features as in Figs 6, 7. Small-medium sized: L: 7.4–8.9 mm; TL: 7.1–8.5 mm.

***Color***: Head, prothorax and pterothorax concolorous dark brown to blackish; mandibles and palpomeres at apex, and antennomeres 1 and 2 dark reddish; following antennomeres markedly darkened at apex and on the dorsal side; abdominal sterna 4–7 blackish, each with a dark reddish spot at sides; elytra dark metallic green, blue green, blue violet or cupreous violet.

***Luster and microsculpture***: head and pronotum glossy, cuticular microlines mostly effaced; elytra moderately glossy, with mesh pattern transverse, and evident microlines.

***Head***: subquadrate, with deep neck constriction; genae swollen, markedly curved and abruptly constricted at the neck, not contiguous with the posterior margin of eyes which are longer than genae and prominent; frons smooth; frontal furrows elongate, with deep longitudinal wrinkles and sparse punctuations; two supraorbital setae on each side.

***Prothorax***: transverse-cordiform, slightly wider than long (ratio PL/PW:0.82), with lateral margins markedly sinuate in the posterior third and constricted to the base. Lateral grooves wide, punctate, widened and flattened in the posterior two/thirds; lateral reflection moderate, evident in the basal fourth. Disc moderately convex, with dense, deep transverse wrinkles. Basal foveae deep, punctate. Anterior angles rounded, fully effaced; basal angles right. Base straight, completely beaded. Paramedial seta at sides absent; basolateral seta on each side present.

***Elytra***: moderately elongate (ratio EL/EW: 1,7), depressed, slightly widened at the apical third; striae impressed, punctate; intervals convex. Post-humeral sinuation very shallow, pre-apical outer callosity slightly distinguishable on interval 8. Apex beaded. Interval 3 with two small discal and one apical setiferous punctures; umbilicate series of 13 large, foveate punctures along stria 8, interrupted in the middle.

***Hind wings***: fully developed.

***Legs***: elongate. Meso- and metatibiae markedly curved; tarsomeres of stout form. Metatarsomeres 1 and 2 flattened, with evident dorsal grooves; metatarsomere 4 deeply bilobed, its lobes markedly widened and rounded at apex. Tarsal claws denticulate, each with five long teeth on the inner side.

***Abdominal sterna***: abdominal sternum VII with one seta on each side in males, two setae in females. Abdominal sternum IX as in Fig. 10C.

***Male genitalia***: median lobe of aedeagus (Fig. 10A, B) very small, stout, moderately curved; apex short, rounded distally. Endophallus with two elongate, bent and basally connected pieces; dorsal piece longer than the ventral one, and more sclerotized.

***Female genitalia***: not examined.

#### Geographical distribution and habitat.

*Calleida anomala* is known so far from the type locality only: Peru, Madre de Dios, Rio Manu, BIOLAT Biological Station, Pakitza, 356 m a.s.l. The specimens of the type series were obtained mostly by insecticidal fog of bamboo, in September and October.

#### Relationships.

*Calleida anomala* is very isolated into the genus owing to some unique and peculiar morphological characters, i.e., the absence of paramedial (anterior) seta at sides of pronotum and the general features of head and prothorax. However, for some features such as the small-medium sized body, and the abdominal sternum VII with one seta on each side in males, two setae in females, it should be related to a large group of species widely distributed mostly in Venezuela and Brazil (many of them undescribed), including *C. similis* Reiche, 1842, *C. subaenea* Mannerheim, 1837, and *C. purpuripennis* Chaudoir, 1872.

### Calleida demathani sp. nov.

Taxon classificationAnimaliaColeopteraCarabidae

2879D356-5A21-5438-8D5D-1180CE93FE96

http://zoobank.org/ED3D93C1-092E-4321-BDD8-559872A87E29

**Figures 11, 12.**
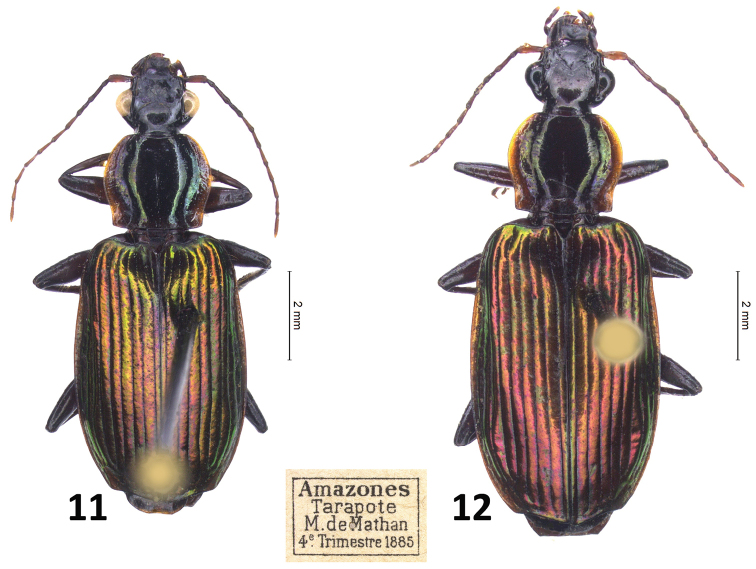
*Calleida demathani* sp. nov. **11** male holotype, habitus (original label in MNHN) **12** idem, female paratype.

Figures 11 , 12 , 14

#### Type locality.

Peru, Tarapoto.

#### Type material.

***Holotype***, male: Pérou, Tarapote (sic), Mai à Août 1886 M. de Mathan (MNHN). ***Paratypes*** (MNHN, NMNH, CCa): 4 ♀♀, same locality and date as holotype; 3 ♂♂, 7 ♀♀ Amazones, Tarapote (sic!), 4e Trimestre 1885 (MNHN, CCA); 2 ♀♀, same locality, Mai à Août 1886 M. de Mathan (MNHN) ; 1 ♀, Pérou, Moyobamba, M. de Mathan, 1^er^ Sem. 1887; 1 ♀, idem, 1888; 1 ♀, Amazones (= Amazonas), S. to Paulo d’Olivença, M. de Mathan [no date] (MNHN). 1 ♂, PERU, MADRE DE DIOS, Rio Manu, BIOLAT Biol. Sta., Pakitza, 356 m 30 Sep 1991 11°56'47"S, 071°17'00"W, T.L. Erwin & M.G. Pogue; Insecticidal fog of Astrocaryum dry leaves (12 fronds) 4 m height Tr. Caña Brava/9 Lot 232; BIOLAT/COLE 000013705; 2 ♀♀, same locality and date, Insecticidal fog of Astrocaryum dry leaves (10 fronds) 4 m height Tr. Caña Brava/9 Lot 226; BIOLAT/COLE 000043497, BIOLAT/COLE 000013496; 1 ♀, same locality and date; Insecticidal fog of Astrocaryum dry leaves (5 fronds) 4 m height Tr. Caña Brava/9 Lot 230 000043497; BIOLAT/COLE 000043497; 1 ♀, same locality, 16 Oct 1991 T.L. Erwin & M.G. Pogue; Insecticidal fog of Astrocaryum dry leaves (10 fronds to 4 m) Tr. Pacal/33 Upper floodplain forest Lot 277; BIOLAT/COLE 000014886; 1 ♀, same locality, 27 Sep 1991 T.L. Erwin; Insecticidal fog of bamboo, green and dry leaves at 2–4 m Tr. Pacal/8 Lot 108; BIOLAT/COLE 000011978; 1 ♂, same locality, 21 Sep 1991 T.L. Erwin; Insecticidal fog of large tree with many lianas and accumulated debris, Tr. Tachigali/9, alluvial terrace forest Lot 91; BIOLAT/COLE 000011838; 1 ♂, same locality, 20 Sep 1991; Insecticidal fog of scattered dry leaves in green, 1 m3, Tr. Tachigali/16.8. dissected alluvial terrace forest Lot 88; BIOLAT/COLE 000012707; 1 ♂, same locality, 12 Feb 90 Erwin/Servat 70°58'W, 12°07'S; Insecticidal fog Tr. Caña Brava/9 tree w/vines and epiphytes: Sloanea; BIOLAT/COLE 000002827; 1 ♂, same locality, 23 Sep 1991 T.L. Erwin; Insecticidal fog of bamboo (2 m3) with dry leaves below green ones, Tr. Zungaro/1 Lot 98; BIOLAT/COLE 00001196; 1 ♂, same locality, 25 Sep 1991 T.L. Erwin; Insecticidal fog of dry leaves at 2.3 m with thin bamboo canopy Tr. Pacal/8 Lot 110; BIOLAT/COLE 0000120009; 2 ♀♀, same locality, Zone 02, 13 Sep 88 T.L. Erwin; Insecticidal fog Canopy/Pouteria BIOLAT 02181013; BIOLAT/COLE 000009221 and BIOLAT/COLE 000009223; 1 ♂, same locality, 09 Sep 88 T.L. Erwin & B.D. Farrell Colls 12°07'S, 70°58W; Insecticidal fog Canopy/Pouteria BIOLAT 02181004; BIOLAT/COLE 000008459; 1 ♀, same locality, 7 Sep 88 T.L. Erwin & B.D. Farrell Colls; Insecticidal fog Canopy/Pourouma sheets 0313 BIOLAT/COLE 000009239; 1 ♀, same locality, 7 Sep 88 T.L. Erwin 11°56'47"S, 071°17'00"W; Insecticidal fog of bamboo, dry leaves at 2 m, plus bamboo canopy at 4–7 m Tr. Pacal/8 Lot 109; BIOLAT/COLE 000011976; 1 ♀, Pakitza zone 03, 13 Oct 89, Erwin/Servat, 70°58'W, 12°07'S; Insecticidal fog Tr. Castanal 42 tree with vines; BIOLAT/COLE 000002811.

#### Specific epithet.

I am very pleased to dedicate this handsome new species to the excellent French explorer and collector of carabid beetles, Marc de Mathan (1876–1908) (for a biographical note, see Moret 1993). He collected in Brazil, Ecuador, Peru, Bolivia, and Panama. More than 130 years ago, he collected a series of specimens of this new species (preserved at the MNHN) in Peru. Several specimens that I attribute to this species were more recently collected at Pakitza, Rio Manu, by T.L. Erwin and co-workers(see below, in Description: notes about the variability).

#### Diagnosis.

With the character states of the genus *Calleida* in the wider sense, but from the closest Neotropical species markedly characterized by the peculiar combination of the following morphological features: medium sized (TL: mm 8.7–10.5); pronotum transverse-cordiform, with lateral furrows very wide, flattened, and lateral margins reflexed and sinuate in the basal fourth; lateral grooves very wide and flattened, deeply punctate and wrinkled; lateral reflection evident. Elytra elongate-ovate, not beaded at apex and with outer apical angle thickened and obtusely rounded; elytral striae deep, punctate, intervals convex. Body markedly bicolored: head blackish, pronotum brown with evident metallic green reflection on disc and lateral margins widely reddish, underside and legs dark brown, contrasting with the metallic purple or purple greenish or green elytra. Abdominal sternum VII with median deep excision and two setae on each side in males (three on one side, exceptionally, in one examined individual), three setae in females. Male genitalia as in Fig. 14.

#### Description.

General features as in Figs 11, 12. Medium sized: L: 9.5–11.2 mm; TL: 8.7–10.5 mm.

***Color***: head black, with two slightly distinct or vanished reddish spots on frons; antennomere 1 reddish, antennomeres 2–4 blackish, following antennomeres reddish, darkened at apex. *Prothorax*, pterothorax, legs, abdomen, basal margin and epipleura of elytra concolorous brown with metallic reflection. Lateral margins of pronotum widely reddish. Elytra on disc bright metallic purple red or red greenish or green with purple reflection.

***Luster and microsculpture***: head, pronotum and elytra glossy, with generally effaced microlines on head and pronotum, more evident on the elytral intervals, in form of isodiametric mesh pattern.

***Head***: wide, flattened, with evident neck constriction; frontal furrows short, wide; genae short, very swollen, not contiguous with the posterior margin of eyes; eyes very large and prominent; two supraorbital setae on each side.

**Figure 13.**
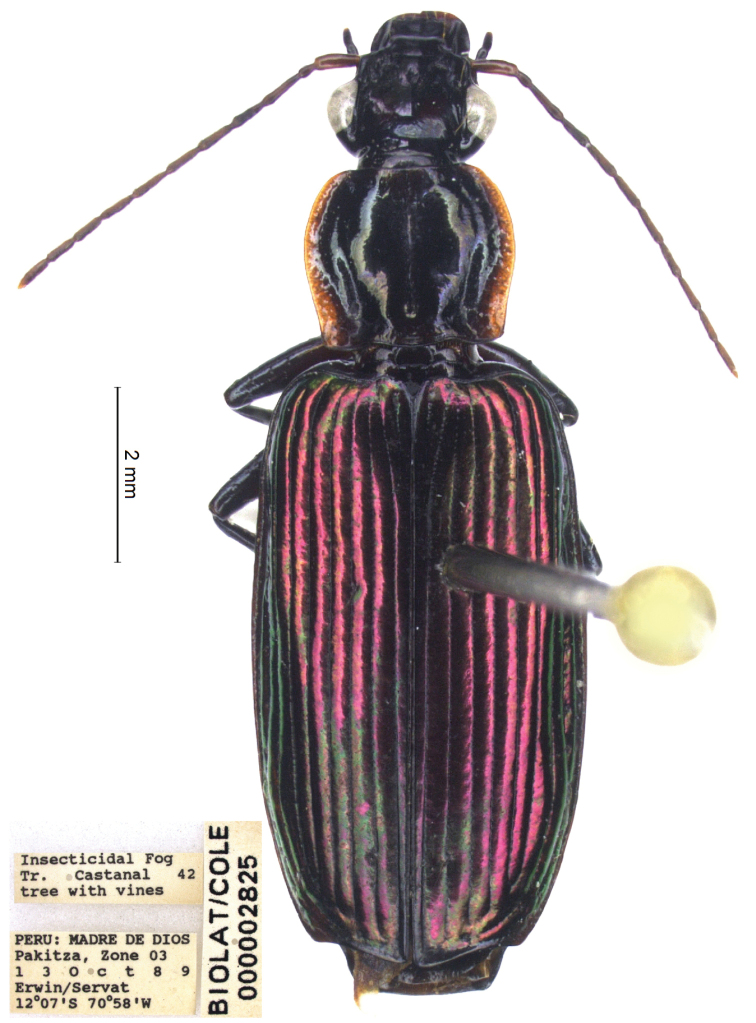
*Calleida erwini* sp. nov., female paratype, habitus.

***Prothorax***: transverse-cordiform (ratio PL/PW: 0.91), with lateral margins widely rounded in the anterior half, in some individuals constricted in front, markedly sinuate in the basal fourth. Lateral grooves very wide and flattened, deeply punctate and wrinkled; lateral reflection evident. Disc depressed, with shallow transverse wrinkles. Median furrow deep, complete, reaching both the anterior and posterior margins; basal foveae deep, wide, almost smooth. Anterior angles rounded, fully effaced; basal angles obtuse. Base oblique at sides, beaded. One paramedial seta and one basolateral seta on each side present.

***Elytra***: moderately elongate (ratio EL/EW: 1.60), slightly widened at the apical third; striae deeply impressed, punctate; intervals convex, with small but evident punctuations. Interval 3 with two discal and one apical setiferous punctures; umbilicate series of 15 punctures along stria 8, interrupted in the middle. Post-humeral sinuation shallow but evident; pre-apical callosity small, distinct on interval 8. Apex not beaded, with outer apical angle thickened and obtusely rounded. Interval 3 with two discal and one apical setiferous punctures; umbilicate series of 14 punctures along stria 8, interrupted in the middle.

***Hind wings***: fully developed.

***Legs***: robust, tarsomeres wide, depressed. Metatarsomeres 1 and 2 shallowly grooved on the dorsal side; metatarsomere 4 deeply bilobed, its lobes widened at apex. Tarsal claws denticulate, each with six long teeth on the inner side.

***Abdominal sterna***: abdominal sternum VII with deep median excision and two setae on each side in males (three on one side, exceptionally, in one examined individual), three setae in females.

**Figures 14–16.**
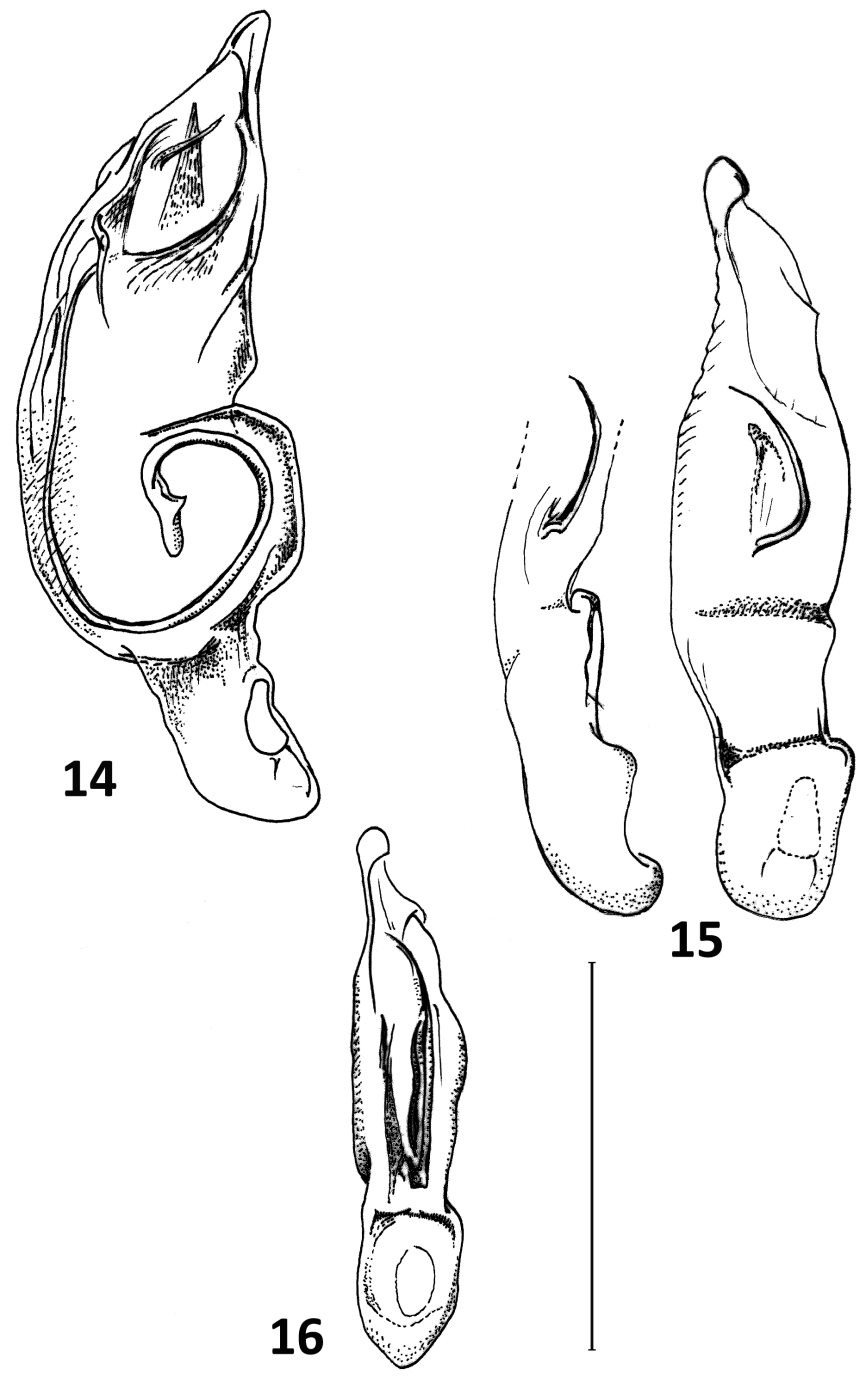
Median lobe of aedeagus of *Calleida* species **14***C. demathani* sp. nov., male holotype **15***C. erwini* sp. nov., male holotype **16***C. marginithorax* sp. nov., male holotype. Scale bar: 1 mm.

***Male genitalia***: median lobe of aedeagus (Fig. 14) relatively large-sized, depressed at sides, widened and ventrally prominent at the basal third; apical lamina obtusely rounded at apex. Endophallus with a long, basally twisted flagellum, and two subapical spines.

***Female genitalia***: not examined.

#### Notes about the variability of the species.

Despite the long distance between Tarapoto (type locality) and Pakitza, there are no evident morphological characters that differentiate specimens collected at Rio Manu from those from Tarapoto.

#### Geographical distribution and habitat.

Widely spread in the western Amazonian basin: south-western Peru: San Martin (Tarapoto) and Madre de Dios regions; Brazil: western Amazonas, S. to Paulo d’Olivença. Individuals were obtained by T. Erwin and co-workers in February, September and October at Rio Manu by fogging canopies of different trees, and in May to August by M. de Mathan in other localities (1885–1888: see Type material).

### Calleida erwini sp. nov.

Taxon classificationAnimaliaColeopteraCarabidae

0204BF88-52B9-59F0-804D-A10AC66D7D8B

http://zoobank.org/31381714-926E-4AE0-B469-C480471EC4C2

Figures 13 , 15

#### Type locality.

Peru, Madre de Dios, Pakitza, 11°55'48"S, 71°35'18"W

#### Type material.

***Holotype***, male (NMNH): “PERU, MADRE DE DIOS, Pakitza Zone 01 21 Sep 89 TL Erwin 11°55'48"S, 71°35'18"W”, “Tr. Tachigali/19 On leaf in webbing of moth”, “BIOLAT/COLE 000011606”. ***Paratypes*** (NMNH, CCa): 1 ♂, 1 ♀, same locality as holotype, Zone 03, 13 Oct 89, Erwin/Servat 12°07"S, 70°58'W, Insecticidal fog Tr. Castanal 42 tree with vines, BIOLAT/COLE 000011537, BIOLAT/COLE 000002825; 1 ♂, PERU, MADRE DE DIOS, Rio Manu, BIOLAT Biol. Sta., Pakitza, 356 m 30 Sep 1991 11°56'47"S, 071°17'00"W, T.L. Erwin & M.G. Pogue, Insecticidal fog of vine tangle at 4 m some green leaves Tr. Tachigali/20.5 Lot 133, BIOLAT/COLE 000012196; 1 ♂, same locality and collectors, 9 Oct 1991, Insecticidal fog of Astrocaryum dry leaves (13 fronds) 4–5 m height Tr. Caña Brava/4 Lot 220, BIOLAT/COLE 000013338.

#### Specific epithet.

I am very pleased to dedicate this new, attractive species to my old friend and colleague Terry Erwin, for his huge contribution to the knowledge of the New World carabids, tropical rainforests and Neotropical biodiversity, and its conservation.

#### Diagnosis.

With the character states of the genus *Calleida* (in the wider sense: see Materials and methods), but from all Neotropical species markedly characterized by the peculiar combination of the following morphological features: medium sized (TL: mm 10.7–11.7); pronotum subquadrate-transverse, widened at base, with lateral margins slightly constricted in front; elytra elongate and narrow, not beaded at apex and with outer apical angle acutely prominent; elytral striae deep, shallowly punctate. Body markedly bicolored: head, pronotum, underside and appendages concolorous brown to blackish, contrasting with the metallic purple or purple red or red greenish elytra. Abdominal sternum VII with one seta on each side in males, two setae in females. Male genitalia as in Fig. 15.

#### Description.

General features as in Fig. 13. Medium sized: L: 11.0–12.0 mm; TL: 10.7–11.7 mm.

***Color***: head dark brown or blackish, with two reddish spots on frons; apex of mouth parts and antennomere 1 reddish; following antennomeres brown reddish, darkened in the apical half. Prothorax, pterothorax (except the lateral margins), legs, abdomen, basal margin and epipleura of elytra concolorous brown to dark brown. Lateral margins of pronotum widely reddish. Elytra on disc metallic purple red or red greenish, with sutural interval, and lateral and apical margins dark metallic green.

***Luster and microsculpture***: head, pronotum and elytra glossy, with generally effaced microlines on head and pronotum, more evident on the elytral intervals, in form of isodiametric mesh pattern.

***Head***: wide, flattened, with evident neck constriction; frontal furrows short, with some deep transverse wrinkles; genae short, markedly swollen, almost contiguous with the posterior margin of eyes; eyes very large and prominent; two supraorbital setae on each side.

***Prothorax***: subquadrate-transverse (ratio PL/PW: 0.9), widened at base, with lateral margins slightly sinuate in the basal half and constricted in front. Lateral grooves wide and flattened, deeply punctate; lateral reflection moderate, evident in the basal fourth. Disc depressed, with shallow transverse wrinkles. Median furrow very deep; basal foveae deep, elongate, smooth. Anterior angles rounded, fully effaced; basal angles obtuse. Base sinuate at sides, beaded. One paramedial seta and one basolateral seta on each side present.

***Elytra***: elongate, narrow (ratio EL/EW: 1.8), sub-convex, slightly widened at the apical third; striae deeply impressed, shallowly punctate; intervals convex. Post-humeral sinuation shallow but evident; pre-apical callosity small, slightly distinct on interval 8. Apex not beaded, deeply sinuate, with both the outer and sutural angles obtusely prominent. Interval 3 with two discal and one apical setiferous punctures; umbilicate series of 12–14 punctures along stria 8, interrupted in the middle.

***Hind wings***: fully developed.

***Legs***: slender, tarsomeres narrow. Metatarsomeres 1–3 grooved on the dorsal side; metatarsomere 4 deeply bilobed, its lobes widened at apex. Tarsal claws denticulate, each with nine long teeth on the inner side.

***Abdominal sterna***: sternum VII with one seta on each side in males, two setae in females.

***Male genitalia***: median lobe of aedeagus (Fig. 15) relatively big-sized, elongate, with wrinkled and markedly lobate ventral margin at the basal third. Endophallus with a short, curved, spine like flagellum in the middle.

***Female genitalia***: not examined.

#### Geographical distribution and habitat.

Known so far from the Rio Manu area. Individuals were obtained in September and October, on leaves or from insecticidal fogging on different plants.

#### Relationships.

Based on several features (general shape of pronotum and elytra, the number and position of setae on abdominal sternum VII and the peculiar structure of the median lobe of aedeagus), *C. erwini* seems to be related to *C. rufocuprea* Chaudoir, 1872, from Eastern Brazil. It is markedly distinct from it by the larger size, the different color pattern (elytra are metallic cupreous in *C. rufocuprea*) and the apical margins of elytra which in *C. erwini* have both the outer and sutural angles obtusely but markedly prominent.

### Calleida marginithorax sp. nov.

Taxon classificationAnimaliaColeopteraCarabidae

E6CFA621-4421-5920-98F5-2EC0998AB058

http://zoobank.org/BF57A13F-8B30-4D04-9134-40BC2B222875

Figures 16 , 17

#### Type locality.

Peru, Madre de Dios: Rio Manu BIOLAT Biol. Sta., Pakitza, 356 m a.s.l., 11°56'47"S, 071°17'00"W.

#### Type material.

***Holotype***, ♂: “PERU, MADRE DE DIOS, Rio Manu, Biolat Biol. Sta., Pakitza, 356 m 10 Oct 1991 11°56'47"S, 71°17'00"W”, T.L. Erwin & M.G. Pogue”; “Insecticidal fog of Astrocaryum dry leaves (5 fronds) 4 height Tr. Caña Brava/9 Lot 230” “BIOLAT/COLE 000013642”. ***Paratypes***: 2 ♀♀, same locality and collectors, 28 Sep 1991” “Insecticidal fog of dry leaves (2 m3) in vines Tr. Tachigali/22 Lot 146”, BIOLAT/COLE 000012479, 12480; 1 ♀, same locality and collectors, 30 Sep 1991, Insecticidal fog of vine tangle at 4 m Some green leaves Tr. Tachingali/20.5, Lot 132; BIOLAT/COLE 000012195; 1 ♂, same locality and collectors, Zone 01, 3 Oct 89, T.L. Erwin & M.G. Pogue; Insecticidal fog canopy Cedrelinga Biolat 03130303; BIOLAT/COLE 000011608; 1 ♂, same locality and collectors, 4 Oct 1991, Insecticidal fog of dry Cecropia leaves in small broadleaf tree at 4 m (1m3) Tr. Tachigali/45 Lot 192, BIOLAT/COLE 000012860; 1 ♂, same locality and collectors, 10 Oct 1991, Insecticidal fog of Astrocaryum dry leaves (12 fronds) 4 height Tr. Caña Brava/4 Lot 232, BIOLAT/COLE 000013722; 1 ♂, same locality and collectors, 10 Oct 1991, Insecticidal fog of Astrocaryum, Lot 229, BIOLAT/COLE 000013622; 1 ♀, same locality and collectors, 14 Oct 1991, Insecticidal fog of Astrocaryum (13 fronds to 4 m) Tr. Pacal/24–25 Lot 267, BIOLAT/COLE 000014578; 1 ♀, same locality, Zone 03, 13 Oct 89, Erwin/Servat 12°07S, 70°58W, Insecticidal fog Tr. Castanal bamboo; BIOLAT/COLE 000010830; 1 ♀, same locality, Zone 02, 06 Sep 89, T.L .Erwin & B.D. Farrell, Insecticidal fog Canopy/Pouteria, BIOLAT/COLE 000001023; 1 ♂, same locality and collectors, Zone 02, Insecticidal fog Canopy/Spondias 02130716, BIOLAT/COLE 000001030; 1 ♀, same locality, 12 Sep 88, T.L. Erwin, 11°55'48"S, 71°35'18"W, Insecticidal fogging demo Biolat 0312, BIOLAT/COLE 000010830; 1 ♂, same locality and collector, 20 Sep 91, Insecticidal fog of scattered dry leaves 1m^3^, Tr. Tachigali/16.8, dissected alluvial terrace forest Lot 88; BIOLAT/COLE 000012706; 1 ♂, same locality and collector, Zone 02, 26 Sep 1991, Insecticidal fog of bamboo at 4 m some green broad leaves under Tr. Zungaro/3.4 Lot 123, BIOLAT/COLE 000012103.

#### Specific epithet.

The specific name stresses the wide, deep lateral furrows of pronotum, which are reddish, markedly contrasting in color with the blackish disc.

#### Diagnosis.

With the character states of the genus *Calleida*, but from the closest Neotropical species markedly characterized by the peculiar combination of the following morphological features: small-medium sized (TL: mm 7.5–8.0); pronotum subquadrate, widened at base, with lateral margins slightly constricted in front, sinuate in the posterior third, and lateral furrows markedly widened and deep; elytra elongate, beaded at apex, with outer apical angle rounded but thickened; elytral striae deep, punctate; intervals convex, finely punctate. Body markedly bicolored: head, pronotum, underside and appendages brown to blackish, contrasting with the metallic purple red or green elytra. Abdominal sternum VII with two setae on each side in males, three to six setae in females. Male genitalia as in Fig. 16.

#### Description.

**Figure 17.**
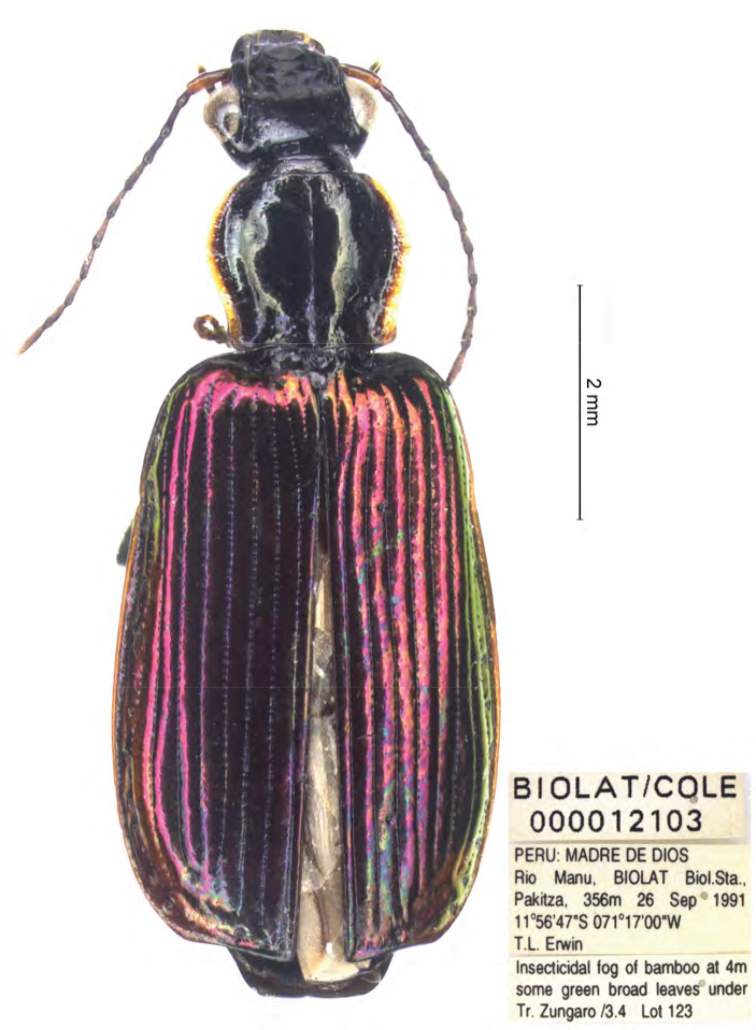
*Calleida marginithorax*, female paratype, habitus.

General features as in Fig. 17. Small-medium sized: L: 8.0–8.5 mm; TL: 7.5–8.0 mm.

***Color***: head blackish, without reddish spots on frons; antennomere 1 light reddish; following antennomeres brown reddish. Prothorax, pterothorax (except the lateral margins), legs, abdominal sterna, basal margin and epipleura of elytra brown to dark brown, the latter with marked metallic green reflection. Lateral margins of pronotum and sides of abdominal sterna reddish. Elytra on disc metallic purple red or red greenish, with lateral margins dark metallic green, or fully metallic green.

***Luster and microsculpture***: head, pronotum and elytra very glossy, with generally effaced transverse microlines on head and pronotum, more evident on the elytral intervals, in form of isodiametric mesh pattern.

***Head***: wide, flattened, with deep neck constriction; frontal furrows deep, short, reaching the anterior supraorbital pore, with some punctuations or longitudinal wrinkles; genae long, markedly swollen, regularly bent, almost contiguous with the posterior margin of eyes; eyes very large, hemispherical, prominent; two supraorbital setae on each side.

***Prothorax***: subquadrate (ratio PL/PW: 0.93), widened at base, with lateral margins slightly sinuate in the basal third and slightly constricted in front. Lateral grooves wide, widened and flattened in the posterior third, punctate; lateral reflection moderate, evident in the basal fourth. Disc sub-convex, smooth or with shallow transverse wrinkles. Median furrow deep, reaching the basal margin; basal foveae deep, obliquely impressed, smooth. Anterior angles rounded, fully effaced; basal angles obtuse. Base oblique at sides, beaded. One paramedial seta and one basolateral seta on each side present.

***Elytra***: moderately elongate (ratio EL/EW: 1.50), sub-convex but with a shallow depression in the basal third, slightly widened at the apical third; striae deep, punctate; intervals convex. Post-humeral sinuation shallow but evident; pre-apical callosity small, slightly distinct on interval 8, or fully vanished. Apex beaded, obliquely truncate, with outer angle rounded, but thickened. Interval 3 with two discal and one apical setiferous punctures; umbilicate series of 13 or 14 large foveate punctures along stria 8, interrupted in the middle.

***Hind wings***: fully developed.

***Legs***: short, robust; tarsomeres narrow. Metatarsomeres 1–3 deeply grooved on the dorsal side; metatarsomere 4 deeply bilobed, its lobes narrow, not widened at apex. Tarsal claws denticulate, each with five long teeth on the inner side.

***Abdominal sterna***: sternum VII with two setae on each side in males, three to six setae in females.

***Male genitalia***: median lobe of aedeagus (Fig. 16) small-sized, moderately elongate, rounded at apex. Endophallus with two long, bent and spine-like copulatory pieces connected at base.

***Female genitalia***: not examined.

#### Geographical distribution and habitat.

Known so far only from the type locality. Individuals were obtained mostly in September and October by insecticidal fog from different species plants.

### Calleida maxima sp. nov.

Taxon classificationAnimaliaColeopteraCarabidae

FCD56EBD-288C-5872-8BAD-B55DC015FBCC

http://zoobank.org/7AE1FFBB-573D-4000-8042-6626855794A4

**Figures 18, 19.**
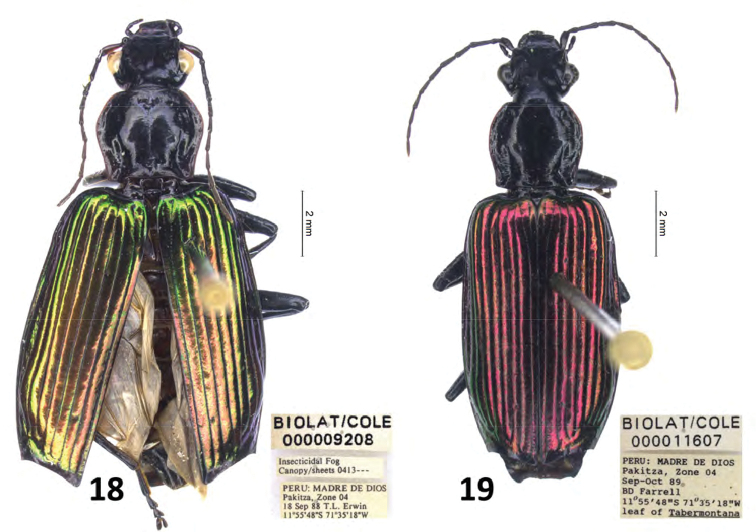
*Calleida* spp. from Rio Manu (Peru), habitus **18***Calleida maxima* sp. nov., female holotype **19***C. jeanneli* Liebke, specimen from Rio Mau (Peru).

**Figures 20–23.**
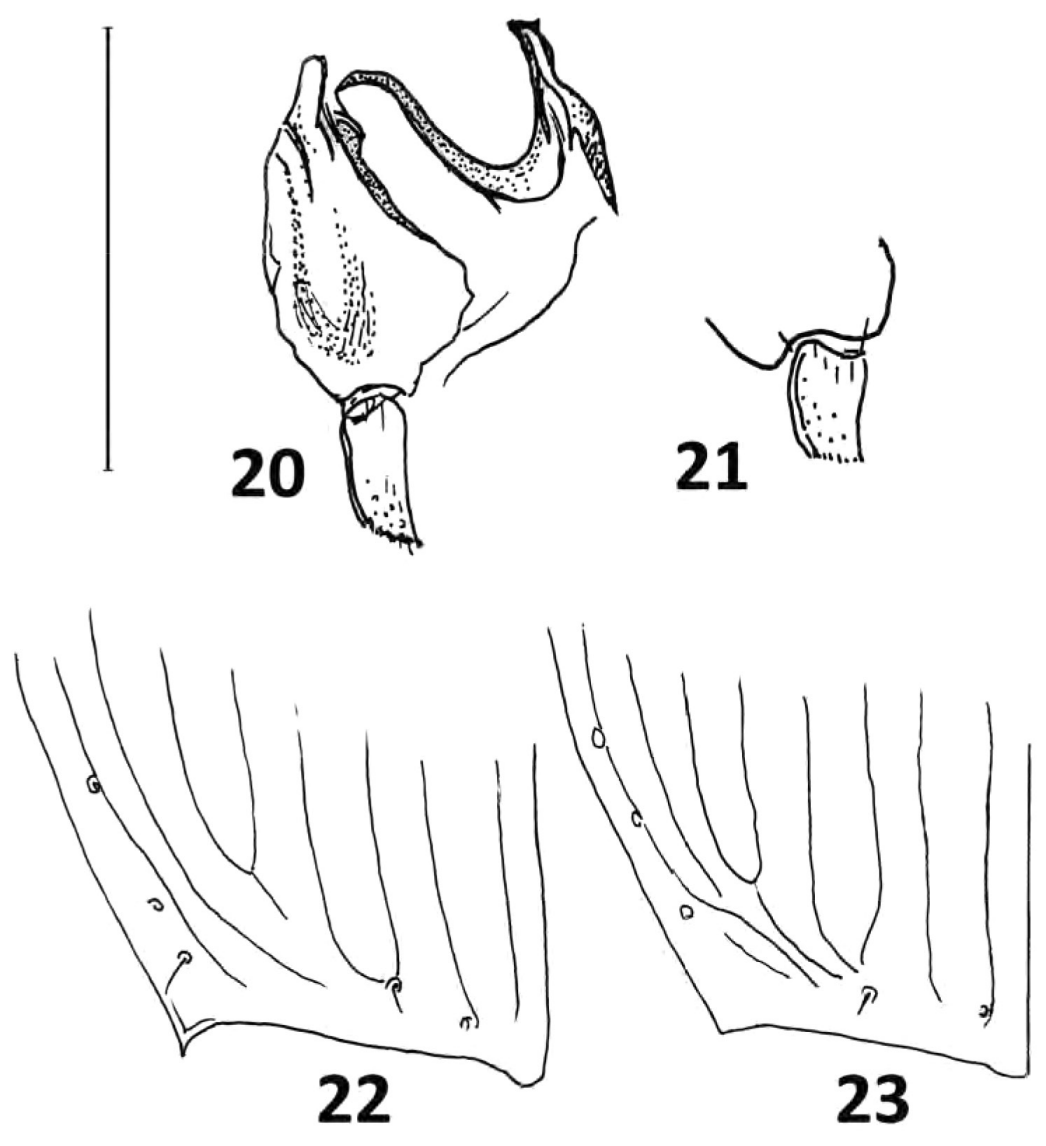
*Calleida* spp. from Rio Manu (Peru), female genitalia (apical reproductive tract and gonocoxite 2) and apical margin of elytra **20***C. maxima* sp. nov. **21***C. jeanneli* Liebke **22***C. maxima* sp. nov. **23***C. jeanneli* Liebke. Scale bars: 0.25 mm (**20, 21**).

Figures 18 , 20 , 22

#### Type locality.

Peru, Madre de Dios, Pakitza, Rio Manu, BIOLAT Biological Station, Pakitza, 356 m a.s.l.

#### Type material.

***Holotype***, ♀: “PERU: MADRE DE DIOS, Pakitza, Zone 04 18 Sep 88 T.L. Erwin 11°55'48"S, 71°35'18"W”, “Insecticidal Fog Canopy/sheets 0413”, “BIOLAT/COLE 000009208”.

#### Specific epithet.

The specific name, *maxima* (superlative of the Latin adjective *magnus, -a, - um*, large in size), indicates the biggest size of this magnificent new species, one of the largest in size of the genus *Calleida*.

#### Diagnosis.

With the character states of the genus *Calleida* (in the wider sense: see Materials and methods), but from the closest Neotropical species markedly characterized by the peculiar combination of the following morphological features: larger in size (TL: mm 14.6), elongate; pronotum moderately transverse, slightly constricted in front; elytra deeply striate, not beaded at apex, with apical external angle acutely prominent, spine-like. Body and appendages uniformly brown blackish, contrasting in color with the metallic green elytra. Abdominal sternum VII with five or six setae on each side in the female (holotype). Female genitalia (ovipositor) as in Fig. 20. Male unknown.

#### Description.

General features as in Fig. 18. Large sized: L: 15.4 mm; TL: 14.6 mm.

***Color***: head black, with two reddish spots on frons; antennomere 1 dark reddish brown, following antennomeres brown blackish. Prothorax, pterothorax, legs, abdomen, basal margin and epipleura of elytra concolorous brown blackish; disc and lateral margins of elytra metallic green with golden reflection.

***Luster and microsculpture***: head and pronotum glossy, with highly effaced microsculpture, hardly visible as transverse mesh pattern; elytra glossy, with fine, hardly visible microlines in form of isodiametric mesh pattern.

***Head***: transverse, dorsally smooth, with moderate neck constriction; genae markedly convex, not contiguous with the posterior margin of eyes; eyes moderately large but very prominent; two supraorbital setae on each side.

***Prothorax***: moderately transverse (female) (ratio PL/PW:0.8); pronotum with lateral margins regularly arcuate, slightly constricted in front, very slightly sinuate anteriorly to the basal angles. Lateral reflection moderate, more evident anteriorly to the basal margin. Disc depressed, with very deep median furrow and very shallow transverse wrinkles. Basal foveae very elongate and deep. Anterior angles rounded, slightly prominent; basal angles obtuse. Basal margin beaded. One paramedial seta and one basolateral seta on each side present.

***Elytra***: moderately elongate (ratio EL/EW: 1.6), depressed; striae impressed, with very shallow punctuations; intervals moderately convex. Post-humeral sinuation shallow, pre-apical callosity evident, markedly prominent. Apex not beaded, deeply hollow, with both sutural and outer angle prominent, the outer spine-like. Interval 3 with two discal and one apical setiferous punctures; umbilicate series of 15 punctures along stria 8, interrupted in the middle.

***Hind wings***: fully developed.

***Legs***: long, slender. Metatarsomere 1 very long, deeply grooved dorsally; metatarsomeres 2 and 3 depressed, with shallow dorsal groove; metatarsomere 4 deeply bilobed, its lobes short, dilated and sub-truncate at apex. Tarsal claws denticulate, each with eight teeth on the inner side.

***Abdominal sterna***: sternum VII with five or six setae on each side in the female (holotype).

***Female genitalia***: (gonocoxites 1 and 2 of ovipositor) as in Fig. 20.

Male unknown.

#### Geographical distribution and habitat.

Known so far only from the type locality. The only female individual was obtained by insecticidal fog canopy, in September.

#### Relationships.

Based on its general features, large size, structure of female genitalia, and the high number of setae on the 7^th^ abdominal sternum, *C. maxima* sp. nov. is close to *C. gigantea* Casale, 2008 from southern Ecuador (Macas) and *C. jeanneli* Liebke, 1935 from Peru. From the latter, sympatric at the Rio Manu river (see Figs 19, 23), it is markedly distinct for its larger size, much wider head, pronotum, and elytra, different color of elytra (which are concolorous metallic purple-red in *C. jeanneli*), the apical outer angle of elytra acutely prominent (Figs 22, 23), and the more elongate gonocoxite 2 of ovipositor (Figs 20, 21). These taxa form a very homogeneous group of highly derived and rare, specialized species, apparently localized to a reduced area between southern Ecuador and Peru, on the Amazon side of the Andes (Casale 2008).

## Concluding remarks

In his contribution to the knowledge of the Rio Manu Area, Erwin (1991) estimated the occurrence of 118 different genera of Carabidae, and for each genus furnished a hypothetical number of species. As a rule in tropical areas, Lebiini are the most numerous representatives: *Calleida* was estimated at 23 species and *Agra* was confirmed as the most diverse and speciose genus, with 44 species. Examination of his material showed that this estimation was almost exact: 22 *Calleida* species of the same genus coexist in sympatry in this area. Some of them are undescribed (seven described in this contribution), and a few, such as *C. jeanneli* Liebke (see Fig. 23) and *C. demathani* sp. nov., also occur in other areas of the Amazon basin. Erwin (1991), again concerning *Calleida*, added that “many new ones are in need of revision, and the numerous species ... will make it a troublesome group to study”.

From a biogeographical standpoint, as in Casale (1998, 2008), Calleidina, and related subtribes of Lebiini, are phyletic lineages of carabids mostly having originated and differentiated in tropical Gondwanan areas with few immigrants (or scarce extant elements) in the Holarctic Region. What about the representatives of the genus Calleida in Peru, and the Pakitza region in particular? In spite of the inadequate knowledge of this genus in the country, the available data show the presence of some phyletic lineages perfectly in agreement with the geographical position of the country, and its exceptional landscape mentioned in the Introduction. At present, some species groups seem to be absent from Peru, such as the *sumptuosa* species group, mostly represented in Central America. Almost all other groups of species are represented in Peru by at least one endemic (precinctive) species.

This datum once again makes evident that this area is one of the main hotspots of biodiversity in the Neotropics, in which several lineages have differentiated and speciated in the Amazon basin, or in the Pacific forests on the western side of the Andes, subsequently overlapping in the country, with some examples of adaptive radiation in sympatric conditions.

The pre-zygotic isolation amongst sibling species in the same area was favored by features of the male and female genitalia, which in Calleidina are highly complex and differentiated, compared to other Lebiini, and very informative from the phylogenetic standpoint. In this lineage, it is possible to show the modification of the median lobe of aedeagus from an elongate, sub-cylindrical, generalized shape, to a depressed, ovate or complex, ventrally lobate shape, and the transformation of the copulatory piece into a bilobed piece, or into a long or very long and twisted flagellum (see Figs 8–10, 14–16). These states have been verified in hundreds of examined and dissected individuals of *Calleida* species, both described and not yet described and demonstrate the occurrence, in this genus, of very homogeneous, markedly distinct to each other species groups, associated with external features, like the number of setiferous punctures in the anal abdominal segment, and other sexually dimorphic characters.

Female genitalia in Calleidina are also very informative (Casale 1998), but have not yet been carefully examined in a sufficiently high number of species. The reproductive tract, in particular, presents markedly modified structures in both spermatheca and spermathecal gland (Casale 2008; Casale and Shi 2018; Shi and Casale 2018). Concerning the ovipositor, it is well known that morphology of gonocoxites in all carabids is tied to different functions (for instance, the telescopic ovipositor of *Agra* may be an adaptation to lay eggs deep in existing burrows in woods or other fissures: Erwin and Pogue 1988). Highly modified and slightly sclerotized gonocoxae are typical of the most derived Lebiini, such as the parasitoid Lebiina, the arboreal Dromiina and Calleidina, and the myrmecophilous Pseudotrechina. Changes in gonocoxite 2 are surely tied to the loss of the digging function of ovipositor. In all Calleidina, the loss of ensiform setae and preapical furrow is assumed as the main synapomorphic feature. In *Calleida*, the modifications of gonocoxite 2 (see Figs 20, 21) are evident, and should indicate different ways of life, and different adaptations of laying eggs on trees and shrubs. We know that *Calleida* species, both as adults and larvae, are actively predatory beetles on caterpillars of small-sized Lepidoptera of different families (Casale and Shi 2018), and Erwin (1991) reported that some of them are specialized canopy dwellers; however, various species are adapted to tree crowns, dry leaf clumps suspended in the undercanopy, understory shrubs, and even tall grasses; most species are diurnal, but a few are nocturnal (e.g., *C. solitaria*) and often fly to lights.

## Supplementary Material

XML Treatment for Calleida solitaria

XML Treatment for Calleida manuensis

XML Treatment for Calleida anomala

XML Treatment for Calleida demathani

XML Treatment for Calleida erwini

XML Treatment for Calleida marginithorax

XML Treatment for Calleida maxima
